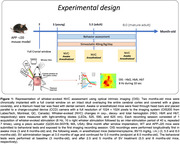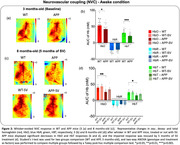# Simvastatin restores neurovascular coupling and cognition in APP mice

**DOI:** 10.1002/alz.091860

**Published:** 2025-01-09

**Authors:** Maria Paula Faccin Huth, Miled Bourourou, Frédéric Lesage, Edith Hamel, Aline R. Zimmer

**Affiliations:** ^1^ Federal University of Rio Grande do Sul, Porto Alegre, Rio Grande do Sul Brazil; ^2^ McGill, Montreal, QC Canada; ^3^ École Polytechnique de Montréal, Montréal, QC Canada; ^4^ Montreal Neurological Institute, Montreal, QC Canada

## Abstract

**Background:**

Alzheimer's disease (AD) is the leading cause of dementia worldwide and vascular dysfunction represents one of the first abnormalities in AD spectrum. Brain imaging techniques that use changes in hemodynamic signals to measure alterations in neurovascular coupling (NVC) have proven useful for early detection of cognitive deterioration. Pharmacological interventions targeting vascular risk factors, including simvastatin (SV), show promise in preventing dementia. To better understand the changes in brain NVC through the AD progression, we measured whisker‐evoked changes in hemodynamics signals and cognition longitudinally in a transgenic mouse model of AD (APP‐J20 mice) and wild‐type (WT) mice treated or not with SV.

**Method:**

Hemodynamic signals following whisker stimulation (8Hz, 10 sec) were recorded longitudinally (3‐8 months) in anesthetized (ketamine/xylazine) and awake WT and APP mice, implanted with full cranial window, using optical imaging of intrinsic signals (OIS) (525, 590, and 625 nm), together with cognitive testing. The effects of SV treatment were evaluated after 2.5 and 5 months.

**Result:**

APP mice displayed significant NVC deficits compared to WT littermates in both awake and anesthetized conditions. The anesthesia worsened the whisker‐evoked hemodynamic responses in APP mice. These changes were detected at early stage of the disease (3 months‐old), before the appearance of cognitive impairment (5.5 months‐old). SV treatment totally restored NVC and cognitive function after 5 months of treatment in APP mice.

**Conclusion:**

The longitudinal analysis of NVC supports their relevance as translational measures for early diagnosis. Our data with SV indicate that preventive cardiovascular strategy for individuals at risk of developing AD may bear promise in protecting brain function.